# Variable-Speed UAV Path Optimization Based on the CRLB Criterion for Passive Target Localization

**DOI:** 10.3390/s25175297

**Published:** 2025-08-26

**Authors:** Lijia Chen, Chengfeng You, Yixin Wang, Xueting Li

**Affiliations:** School of Aeronautics and Astronautics, Sichuan University, Chengdu 610207, China; 2023141510131@stu.scu.edu.cn (L.C.); 2023141510127@stu.scu.edu.cn (C.Y.); 2023141510130@stu.scu.edu.cn (Y.W.)

**Keywords:** target passive localization, unmanned aerial vehicles (UAVs), Cramer–Rao lower bound (CRLB), path optimization method, variable speed, particle swarm algorithm (PSO)

## Abstract

The performance of passive target localization is significantly influenced by the positions of unmanned aerial vehicle swarms (UAVs). In this paper, we investigate the problem of UAV path optimization to enhance the localization accuracy. Firstly, a passive target localization signal model based on the time difference of arrival (TDOA) algorithm, which is then improved by the Chan method and Taylor series expansion, is established. Secondly, the Cramer–Rao lower bound (CRLB) of the modified TDOA algorithm is derived and adopted as the evaluation criterion to optimize the UAVs’ positions at each time step. Different from the existing works, in this paper, we consider the UAVs to have variable speed; therefore, the feasible region of the UAVs’ positions is changed from a circle into an annular region, which will extend the feasible region, enhancing the localization accuracy while increasing the computation complexity. Thirdly, to improve the efficiency of the UAV path optimization algorithm, the particle swarm optimization (PSO) algorithm is applied to search for the optimal positions of the UAVs for the next time step. Finally, numerical simulations are conducted to verify the validity and effectiveness of the proposals in this paper.

## 1. Introduction

As a critical component of modern intelligent unmanned systems, UAVs are profoundly reshaping combat paradigms and technological frameworks in the military domain. In modern warfare, UAVs demonstrate remarkable advantages in key missions such as target reconnaissance [1], battlefield surveillance [2,3], and formation control through their highly coordinated operations. This collaborative combat system transcends limitations of traditional fixed infrastructure, resolves challenges like communication latency [4], and significantly enhances the timeliness of battlefield information acquisition and operational effectiveness [5]. Particularly in complex and dynamic combat environments, UAVs have emerged as critical penetration forces against enemy defense systems due to their high mobility [6,7], strong survivability, and autonomous coordination capabilities [8]. However, most existing studies assume uniform motion for path optimization, which poses major challenges in real-world variable-speed scenarios.

In various combat applications of UAVs, passive target localization technology holds unique tactical value [9,10]. Compared to active localization systems, passive localization systems achieve precise localization of emitter targets without exposing their own location by passively receiving electromagnetic signals from radiation sources [11], playing a crucial role in electronic reconnaissance, anti-stealth operations, and battlefield situational awareness. Tua A. Tamba et al. developed a distributed Kalman filter-based cooperative tracking algorithm for multiple UAVs, which enhances target state estimation accuracy in maritime moving vessel monitoring scenarios. However, their constant-velocity motion model demonstrates limited adaptability to abruptly maneuvering targets [12]. The DQN-ALNS framework proposed by Lingjie Zhou et al. significantly improves the solution efficiency of UAV path optimization through the collaborative optimization of deep reinforcement learning and adaptive large neighborhood search. However, its state space design based on the uniform speed assumption limits its application in dynamic environments [13]. Therefore, how to construct a dynamic geometric constraint model that accommodates variable-speed motion has become the core issue in improving passive localization accuracy.

### 1.1. Overview

For the application of target passive localization based on UAVs, the optimization of data quality [14,15], formation configuration [16] and flight paths significantly impacts the performance of the localization accuracy. By elevating data quality and selecting optimal measurements, the precision and robustness of the localization system can be effectively improved. The bearing-only passive localization model proposed by Ruiqing Yang et al. improves localization accuracy through the fusion of triangulation and greedy strategies, but fails to account for the impact of variable-speed motion on the observation geometric configuration [17]. Through optimized formation geometry, the UAVs can maintain the most effective configuration during target localization, enabling continuous optimal localization geometry that significantly enhances both accuracy and continuity of the localization performance. Meanwhile, UAV optimization involves dynamically adjusting the flight paths and spatial configurations of the UAVs to optimize the observation conditions, ultimately achieving high-precision localization accuracy. These studies ensure that the observational data provided by the UAVs consistently approximates the minimum error conditions, thereby substantially improving the localization performance.

At the path optimization algorithm level, swarm intelligence algorithms have made significant progress, but several critical challenges remain. Zhanghan Li et al. proposed the APF-DDQN model, which integrates artificial potential fields with deep reinforcement learning to effectively address path optimization in complex obstacle-rich environments. However, its state space design does not account for dynamic velocity variations [18]. The improved ACO algorithm proposed by Dong et al. [19] introduces roulette wheel selection and elitist strategies, significantly enhancing task execution efficiency and survival probability in multi-task scenarios while resolving the poor convergence issues of traditional methods in handling multi-constraint, nonlinear optimization problems. The PSO algorithm modified by Hao et al. [20] features nonlinear inertia weight adjustments and incorporates logarithmic function control mechanisms with Gaussian random adjustment strategies, substantially improving convergence speed and local search capabilities in UAV path optimization. Han et al. [21] enhanced multi-UAV cooperative mission optimization by integrating Dubins curve constraints with multi-objective optimization strategies, improving convergence rates and global optimization capabilities while resolving the challenge of simultaneously satisfying trajectory length, mission payoff, and flight constraints. Shengxi Li et al. proposed a hybrid path optimization framework integrating Model Predictive Control (MPC) with Control Barrier Functions (CBFs). While this approach enhances dynamic obstacle avoidance through safety constraints, its prediction model still relies on the uniform motion assumption, resulting in path tracking errors during sudden maneuvering scenarios [22]. However, the modified PSO algorithm only adopts the fixed speed updates [20], which cannot accommodate variable-speed UAV movements. When combined with Dubins curves, the system remains constrained by curvature limitations, confining the search space to a fixed circle, restricting the improvement of the localization accuracy.

For UAV path optimization in complex obstacle-laden environments where computational demands are high and efficiency is low, several algorithms have made breakthroughs. Wang et al.’s priority-based Artificial Potential Field (APF) algorithm [23], Zhang et al.’s improved target-biased Rapidly-exploring Random Tree (RRT) path optimization algorithm [24], Lu et al.’s RRT-APF hybrid path optimization algorithm [25], and Xu et al.’s Three-phase Alternating Optimization Algorithm (TAOA) [26] all overcome these limitations and resolve the issue of excessive computational burdens in complex environments. However, these methods suffer from poor adaptability and are unsuitable for variable-speed environments.

In dynamic environments, Hou et al. proposed a dynamic path optimization algorithm integrating improved Q-Learning with the Artificial Potential Field (APF) method [27], resolving the issue where traditional methods fail to generate effective paths between path nodes. Zhou et al. developed a UAV TDOA localization path optimization algorithm based on the D-optimality criterion [28], significantly enhancing localization accuracy and tracking stability for emitter targets. Li et al. presented a hybrid TDOA/FDOA-based UAV path optimization algorithm [29], substantially improving position and velocity estimation accuracy for moving targets. However, these methods exhibit poor real-time performance and do not consider the situation where the UAVs have variable speed.

To improve the UAV path optimization efficiency, Pan et al. resolved the issues of traditional APF methods easily falling into local minima and formation instability caused by introducing rotation [30]. Sun et al. proposed a UAV path optimization algorithm based on semantic maps and multi-scale feature fusion [31], which notably improves path optimization efficiency for large-scale formations. Additionally, Zhou et al. significantly improved the efficiency and quality of image acquisition for 3D reconstruction through intelligent viewpoint selection and optimization strategies [32]. Chen et al. transformed task allocation problems into a Vehicle Routing Problem (VRP) model and optimized observation time [33], notably increasing the efficiency of multi-UAV cooperative remote sensing missions. Li et al. addressed the low efficiency of traditional swarm collision avoidance optimization by establishing a coupling matrix and employing the AR-SAS path optimization method [34]. Li et al. also studied how to optimize UAV observation geometry configurations [35], significantly reducing the flight path lengths and computational loads while resolving the inefficiency of conventional passive localization methods.

However, the above approaches are all under the assumption that the UAVs have a fixed speed and this will set strict constraints on the UAV path optimization problem, restricting the feasible region to circles with the UAVs as their centers. However, in the real situation, UAVs generally have variable speed; thus, the feasible region of the UAV path optimization problem is an annular region which extends the feasible region, leading to more optimal UAV position solutions.

### 1.2. Original Contribution

Evidently, the existing works mostly focus on UAV path optimization with fixed speed, which is mismatched with the real situation and will restrict the improvement of passive target localization accuracy. Thus, in this paper, we focus on the problem of UAV path optimization, considering UAVs with variable speed. Firstly, the signal model of the passive target localization method is established. Secondly, a UAV path optimization method is proposed based on the CRLB of the TDOA localization method, considering UAVs with variable speed. Thirdly, the PSO algorithm is applied to accelerate the optimization process and improve the efficiency of the path optimization algorithm. Simulation results illustrate that the proposals in this paper could achieve better localization accuracy with less computational complexity. The original contributions of this paper are as follows:

(1) A novel UAV path optimization method based on the CRLB of the TDOA localization method is proposed, where the trace of the CRLB is set as the objective function and UAV moving constraints are set as the constraint conditions. By optimally configuring the positions of the UAVs for each time step, the localization accuracy is improved.
(2) Different from the existing works where the UAVs are assumed to have fixed speed, in this paper, we consider the UAVs to have variable speed; thus, the feasible region of the UAV positions is extended from a circle to an annular region and the localization accuracy is improved.
(3) To accelerate the UAV path optimization process and improve the efficiency of the algorithm, the PSO algorithm is applied to search for the optimal positions of the UAVs at the next time step.

## 2. Signal Model of Passive Target Localization

In this section, the TDOA algorithm is applied for target localization. Since the TDOA algorithm generally has high computation complexity, the Taylor series expansion algorithm is introduced to accelerate the localization algorithm, and the modified TDOA–Taylor algorithm could achieve better computation efficiency. Although the modified TDOA–Taylor algorithm benefits from the smaller computation load, it is affected by the initial value of the target state error at a large scale. If the initial error of the target state is large, the accuracy of the target localization would be poor. Thus, the Chan algorithm is also introduced to guarantee the accuracy of the initial value of the target state, and the modified TDOA–Chan–Taylor algorithm could achieve better localization accuracy with low computation complexity.

### 2.1. Signal Model of the TDOA Algorithm

TDOA refers to a localization technique that determines the location of a target by measuring the time differences of the target’s arrival at different base stations. It is a multi-sensor localization technology which requires at least three receivers for localization.

The distance $r_{j}$ from the target to any base station *j* is

(1) $$r_{j}=\sqrt{{\left(x-x_{j}\right)}^{2}+{\left(y-y_{j}\right)}^{2}}$$

where the target position $u={\left[x,y\right]}^{T}$ and the coordinates of the base station *j* are $\left(x_{j},y_{j}\right)$.

The distance difference $r_{j,0}$ between the target of the secondary station *j* and the primary station is

(2) $$r_{j,0}=\sqrt{{\left(x-x_{j}\right)}^{2}+{\left(y-y_{j}\right)}^{2}}-\sqrt{{\left(x-x_{0}\right)}^{2}+{\left(y-y_{0}\right)}^{2}}$$

Then, we can get the following nonlinear hyperbolic equations:

(3) $$\left\{\begin{array}{c} r_{1,0}=\sqrt{{\left(x-x_{1}\right)}^{2}+{\left(y-y_{1}\right)}^{2}}-\sqrt{{\left(x-x_{0}\right)}^{2}+{\left(y-y_{0}\right)}^{2}}\\ r_{2,0}=\sqrt{{\left(x-x_{2}\right)}^{2}+{\left(y-y_{2}\right)}^{2}}-\sqrt{{\left(x-x_{0}\right)}^{2}+{\left(y-y_{0}\right)}^{2}} \end{array}\right.$$

Thus, the TDOA localization problem reduces to solving for the intersection point of the aforementioned hyperbolic equations.

### 2.2. Modified TDOA–Chan Algorithm

The basic idea of the Chan algorithm is to calculate the position of the target using a two-step weighted least squares algorithm. Instead of the iterative process of the TDOA algorithm, the TDOA–Chan algorithm could obtain the analytical expression solution of the target position, achieving lower computation complexity and a good initial value of the target state.

By squaring the two sides of Equation (1), we get

(4) $$r_{j}^{2}={\left(x-x_{j}\right)}^{2}+{\left(y-y_{j}\right)}^{2}=C_{j}-2x_{j}x-2y_{j}y+x^{2}+y^{2}$$

where $C_{j}=x_{j}^{2}+y_{j}^{2}$.

By arranging and squaring the two sides of Equation (2), we obtain

(5) $$C_{j}-\left(r_{j,0}^{2}+2r_{j,0}r_{0}+r_{0}^{2}\right)=2x_{j}x+2y_{j}y-x^{2}-y^{2}$$

Let j=0; we get

(6) $$C_{0}-r_{0}^{2}=2x_{0}x+2y_{0}y-x^{2}-y^{2}$$

Subtracting Equation (6) from Equation (5), we have

(7) $$2\left(x_{j}-x_{0}\right)x+2\left(y_{j}-y_{0}\right)y=C_{j}-C_{0}-\left(r_{j,0}^{2}+2r_{j,0}r_{0}\right)$$

According to the above derivations, when the total number of the base stations in a two-dimensional space is 3, the relationship between the target position $u={\left[x,y\right]}^{T}$ and $r_{0}$ can be obtained as follows:

(8) $$\left[\begin{array}{c} x\\ y \end{array}\right]={\left[\begin{array}{cc} x_{10} & y_{10}\\ x_{20} & y_{20} \end{array}\right]}^{-1}\times \left\{\left[\begin{array}{c} r_{1,0}\\ r_{2,0} \end{array}\right]r_{0}+\frac{1}{2}\left[\begin{array}{c} r_{1,0}^{2}-C_{1}+C_{0}\\ r_{2,0}^{2}-C_{2}+C_{0} \end{array}\right]\right\}$$

where $x_{j0}=x_{j}-x_{0},\;y_{j0}=y_{j}-y_{0}$.

When the number of the base stations n≥3, a two-step weighted least squares method needs to be applied to obtain the position of the target.

Let the unknown variable matrix $p={\left[u,r_{0}\right]}^{T}$ and the time difference noise vector ζ be

(9) ζ=η−Dp

where

(10) $$\eta =\frac{1}{2}\left[\begin{array}{c} r_{1,0}^{2}-C_{1}+C_{0}\\ r_{2,0}^{2}-C_{1}+C_{0}\\ \vdots \\ r_{(n-1),0}^{2}-C_{1}+C_{0} \end{array}\right]$$

(11) $$D=\left[\begin{array}{ccc} x_{10} & y_{10} & r_{1,0}\\ x_{20} & y_{20} & r_{2,0}\\ \vdots & \vdots & \vdots \\ x_{(n-1),0} & y_{(n-1),0} & r_{(n-1),0} \end{array}\right]$$

Then the covariance matrix ξ of the time difference noise vector ζ is

(12) $$\xi =E\left[\zeta \zeta^{T}\right]=c^{2}ARA^{T}$$

where $A=\mathrm{diag}(r_{0},r_{1},\ldots ,r_{n-1})$ and *R* is the covariance matrix of the time difference measurements.

When the distance is large, the maximum likelihood estimation of p can be written as

(13) $$p\approx {\left(D^{T}\xi^{-1}D\right)}^{-1}D^{T}\xi^{-1}\eta$$

Then we can get

(14) $$p={\left[\hat{x},\hat{y},{\hat{r}}_{0}\right]}^{T}$$

Next, we conduct the calculation of the second weighted least squares algorithm. When there is random noise, *D*, η, and p can be expressed as

(15) $$\left\{\begin{array}{c} D=D^{o}+\Delta D\\ \eta =\eta^{o}+\Delta \eta \\ p=p^{o}+\Delta p \end{array}\right.$$

where $D^{o}$ represents the ideal design matrix under noiseless conditions, $\eta^{o}$ denotes the true observation vector without measurement errors, $p^{o}$ is the true 3D vector containing target coordinates (x, y) and the distance between the target and the reference station, ΔD is the design matrix deviation from base station coordinate measurement errors, Δη is the observation vector error propagated from time difference measurement noise, and Δp is the estimation error of the first weighted least squares step solution.

Then the covariance matrix of Δp is

(16) $$\mathrm{cov}\left(\Delta p\right)=E\left[\Delta p\Delta p^{T}\right]={\left(D^{oT}\xi^{-1}D^{o}\right)}^{-1}$$

Then the expression of the time difference noise vector $\zeta^{\prime }$ can be obtained as

(17) $$\zeta^{\prime }=\eta^{\prime }-D^{\prime }p^{\prime }$$

where $\eta^{\prime }=\frac{1}{2}\left[\begin{array}{c} {\left(\hat{x}-x_{0}\right)}^{2}\\ {\left(\hat{y}-y_{0}\right)}^{2}\\ {\hat{r}}_{0}^{2} \end{array}\right],\;D^{\prime }=\left[\begin{array}{cc} 1 & 0\\ 0 & 1\\ 1 & 1 \end{array}\right]$, $p^{\prime }={\left[x^{\prime },y^{\prime }\right]}^{T}$, $x^{\prime }\approx {\left(x-x_{0}\right)}^{2}$, $y^{\prime }\approx {\left(y-y_{0}\right)}^{2}$.

Following an analogous procedure to Equation (12), the covariance matrix $\xi^{\prime }$ of the time difference noise vector $\zeta^{\prime }$ for the second weighted least squares step can be derived. When the measurement noise and estimation residuals are small, since $\zeta^{\prime }$ obeys the Gaussian distribution, the maximum likelihood estimation of $p^{\prime }$ can be obtained as $p^{\prime }\approx {\left(D^{\prime T}\xi^{\prime -1}D^{\prime }\right)}^{-1}D^{\prime T}{\xi^{\prime }}^{-1}\eta^{\prime }$. When the distance between the base station and the target is large, the covariance matrix of $p^{\prime }$ can be simplified as

(18) $$\mathrm{cov}\left(p^{\prime }\right)\approx {\left(D^{\prime T}\xi^{\prime -1}D^{\prime }\right)}^{-1}$$

According to the above derivations, the unambiguous solution of the unknown quantity p can be obtained through two weighted least squares methods. Finally the position of the target $u={\left[x,y\right]}^{T}$ can be obtained.

### 2.3. Modified TDOA–Chan–Taylor Algorithm

The Taylor expansion algorithm is significantly affected by the initial target position estimate. Thus, this subsection introduces the modified TDOA–Chan–Taylor algorithm. We utilize the target position estimate (x0,y0) of the TDOA–Chan algorithm as the initial value for Taylor expansion. By neglecting second-order and higher terms, Equation (1) is transformed into

(19) ε=h−Gp

where $h=\left[\begin{array}{c} r_{1,0}-\left(R_{1}-R_{0}\right)\\ r_{2,0}-\left(R_{2}-R_{0}\right)\\ \vdots \\ r_{(n-1),0}-\left(R_{n-1}-R_{0}\right) \end{array}\right]$, $G=\left[\begin{array}{cc} \frac{x_{0}-x0}{R_{0}}-\frac{x_{1}-x0}{R_{1}} & \frac{y_{0}-y0}{R_{0}}-\frac{y_{1}-y0}{R_{1}}\\ \frac{x_{0}-x0}{R_{0}}-\frac{x_{2}-x0}{R_{2}} & \frac{y_{0}-y0}{R_{0}}-\frac{y_{2}-y0}{R_{2}}\\ \vdots & \vdots \end{array}\right]$, ε is the residual vector, and $R_{j}$ represents the distance from the *j*-th base station to the initial value.

Using the weighted least squares algorithm, the least squares estimation of the target position can be obtained as follows:

(20) $$p={\left(G^{T}Q^{-1}G\right)}^{-1}G^{T}Q^{-1}h$$

where *Q* is the covariance matrix of the TDOA measurement.

## 3. CRLB Derivation and Problem Formulation

In this section, the CRLB for the TDOA–Chan–Taylor localization algorithm is derived, giving the theoretical lower bound of the target localization accuracy. Then, the trace of the CRLB is set as the objective function with the UAV motion constraints set as the constraint conditions, and the UAV path optimization problem is formulated.

### 3.1. CRLB Derivation for the TDOA Localization Algorithm

For an unbiased estimator, the minimum error of the estimator can be characterized by the CRLB of the estimator. Thus, in this paper, the CRLB of the TDOA algorithm is set as the objective function of the UAV path optimization problem, and through searching for the optimal UAV positions, the accuracy of the target localization performance can be improved.

Based on the TDOA $\tau_{j}$ between the base station *j* and the reference station, we establish the measurement equation as

(21) V=l(u)+m

where $l\left(u\right)={\left[c\tau_{1},\ldots ,c\tau_{n-1}\right]}^{T}$, the measurement error m satisfies the normal distribution $N(\theta_{\left(n-1\right)},R_{\left(n-1)\times (n-1\right)})$, and V is the actual observed value. $\theta_{\left(n-1\right)}$ is a zero-mean vector of dimension (n−1)×1, and R is the measurement noise covariance matrix of dimension (n−1)×(n−1).

According to $u={\left[x,y\right]}^{T}$, we can get the Jacobi matrix $J={\left[\frac{\partial l(u)}{\partial x},\frac{\partial l(u)}{\partial y}\right]}^{T}$.

(22) $$\left\{\begin{array}{c} \frac{\partial l(u)}{\partial x}=\left[\frac{x-x_{1}}{r_{1}}-\frac{x-x_{0}}{r_{0}},\frac{x-x_{2}}{r_{2}}-\frac{x-x_{0}}{r_{0}},\ldots ,\frac{x-x_{n-1}}{r_{n-1}}-\frac{x-x_{0}}{r_{0}}\right]\\ \frac{\partial l(u)}{\partial y}=\left[\frac{y-y_{1}}{r_{1}}-\frac{y-y_{0}}{r_{0}},\frac{y-y_{2}}{r_{2}}-\frac{y-y_{0}}{r_{0}},\ldots ,\frac{y-y_{n-1}}{r_{n-1}}-\frac{y-y_{0}}{r_{0}}\right] \end{array}\right.$$

Then, the Fisher matrix can be obtained as

(23) $$F=J^{T}{\left(R\right)}^{-1}J$$

Taking the inverse of the above equation, the CRLB Cr is obtained:

(24) $$\mathrm{Cr}={\left(F\right)}^{-1}$$

### 3.2. Problem Formulation

In this subsection, the UAV path optimization problem is formulated. Firstly, the minimum trace of the CRLB for the TDOA–Chan–Taylor localization algorithm is set as the objective function of the UAV path optimization problem, which would guarantee the accuracy of the target localization performance. The objective function is shown as follows:

(25) $$\min_{u_{1},u_{2},\ldots ,u_{n}}\mathrm{Tr}\left(\mathrm{Cr}(u_{1},u_{2},\ldots ,u_{n})\right)$$

where $u_{i}$ represents the positions of the *i*th UAV.

Meanwhile, to ensure the feasibility and stability of the UAV flight operations, the following constraint conditions need to be satisfied:

(1) Minimum approach distance constraint: At any given time *t*, the distance $r_{i}^{t}$ from the *i*th UAV to the target needs to satisfy
  (26) $$r_{i}^{t}\geq d_{\min}$$
  where $d_{\min}$ is the minimum allowable distance between the UAVs and the target. This constraint prevents the UAVs from approaching the target too closely and being detected, thus ensuring the stealth and security of the mission.
(2) Heading angle deviation constraint: For the *i*th UAV at time *t*, the heading angle deviation $\phi_{i}^{t}=\psi_{i}^{t+1}-\psi_{i}^{t}$ between two successive time steps must satisfy
  (27) $$\phi_{\min}\leq \phi_{i}^{t}\leq \phi_{\max}$$
  where $\phi_{\min}$ is the minimum value of the heading angle deviation for the UAVs, and $\phi_{\max}$ is the maximum value of the heading angle deviation for the UAVs.
(3) Heading angle constraint: The heading angle $\psi_{i}^{t}$ between the heading angle of the *i*th UAV at time *t* and the reference direction must satisfy
  (28) $$\psi_{\min}\leq \psi_{i}^{t}\leq \psi_{\max}$$
  where $\psi_{\min}$ is the minimum value of the heading angle for the UAVs, and $\psi_{\max}$ is the maximum value of the heading angle for the UAVs. For target localization application, drastic changes in heading deviation angles $\phi_{i}^{t}$ and heading angles $\psi_{i}^{t}$ are detrimental to the UAVs’ successive target observation. Simultaneously, these angular constraints effectively prevent invalid solutions and enhance the efficiency of the path optimization.
(4) Velocity constraint: The velocity $v_{i}^{t}$ of the *i*th UAV at time *t* satisfies
  (29) $$v_{\min}\leq v_{i}^{t}\leq v_{\max}$$
  where $v_{\min}$ is the minimum velocity of the *i*th UAV, and $v_{\max}$ is the maximum velocity of the *i*th UAV.
(5) Energy Constraint: At any given time *t*, the distance $r_{i}^{t}$ from the *i*th UAV to the target needs to satisfy
  (30) $$r_{i}^{t}\leq s_{\max}$$
  (31) $$s_{\max}=v_{\max}\cdot t_{\mathrm{bat}}$$
  where $s_{\max}$ is the maximum allowable maneuver distance of the *i*th UAV and $t_{\mathrm{bat}}$ is the effective battery endurance time. This constraint limits the maximum maneuverable distance of UAVs to ensure that their path planning remains within the battery’s endurance range, thereby preventing mission failure due to power depletion.

Different from the existing works, the UAVs in this paper are considered with variable speed, and the velocity of the UAVs is limited to the range of [$v_{\min}$, $v_{\max}$]. Thus, the feasible region of the optimization problem is extended from the conventional fixed-radius circumferences to annulars centered at the UAVs’ current positions with the inner radius of $v_{\min}$ and the outer radius of $v_{\max}$.

By considering the UAVs with variable speed, the proposals in this paper could be more in accordance with the actual situation, significantly improving the UAVs’ flexibility and environmental adaptability.

## 4. PSO Algorithm

In this paper, the PSO algorithm is applied to search for the optimal UAV positions at each time step to improve the target localization accuracy. Through optimizing the UAV positions for successive time steps, the optimal UAV path would be obtained.

Assume that at each iteration there are *S* particles, and each particle *s* represents a candidate UAV heading angle deviations strategy

(32) $$\eta^{t,k(s)}={\left[\begin{array}{cccc} \phi_{1}^{t,k(s)} & \phi_{2}^{t,k(s)} & \ldots & \phi_{i}^{t,k(s)} \end{array}\right]}^{T}$$

where $\phi_{i}^{t,k(s)}$ represents the heading angle deviation of the *s*th particle at the *k*th iteration of time step *t* for the *i*th UAV. Through the heading angle deviation $\eta^{t,k(s)}$, the position of the *s*th particle at the *k*th iteration of time step *t*, $x^{t,k(s)}$, can be obtained.

At the *k*th iteration of time step *t*, the velocity of the *s*th particle is denoted as $v^{t,k(s)}$ and its heading angle deviation is $\eta^{t,k(s)}$. Then at the (k+1)th iteration of time step *t*, the velocity $v^{t,k+1(s)}$ and heading angle deviation $\eta^{t,k+1(s)}$ of the *s*th particle can be updated as

(33) $$v^{t,k+1(s)}=v^{t,k(s)}+c_{1}r_{1}\left(p^{t,k(s)}-\eta^{t,k(s)}\right)+c_{2}r_{2}\left(p_{g}^{t,k}-\eta^{t,k(s)}\right)$$

(34) $$\eta^{t,k+1(s)}=\eta^{t,k(s)}+v^{t,k+1(s)}$$

where $p^{t,k(s)}$ represents the individual historical best position of the *s*th particle at the *k*th iteration of time step *t*, $p_{g}^{t,k}$ represents the global best position of the entire particle swarm at the *k*th iteration of time step *t*, and $r_{1}$ and $r_{2}$ represent the uniformly distributed random variables in the range of [0,1], which are used to introduce randomness and avoid getting trapped in local optima. $c_{1}$ denotes the individual learning factor, which can adjust the step size of the particle moving towards its own historical best position $p^{t,k(s)}$, and $c_{2}$ denotes the social learning factor, which can adjust the step size of the particle moving towards the global best position $p_{g}^{t,k}$.

To select the optimal solution, the performance of these *S* particles is evaluated using the fitness function. At the *k*th iteration of time step *t*, the fitness function for the *s*th particle is as follows:

(35) $$Fn\left(x^{t,k(s)}\right)=\mathrm{Tr}\left(\mathrm{Cr}\left(x^{t,k(s)}\right)\right)$$

where $Fn(x^{t,k(s)})$ is the fitness value when the heading angle deviation of the *s*th particle is $\eta^{t,k(s)}$ and the related position of the *s*th particle is $x^{t,k(s)}$, $\mathrm{Cr}(x^{t,k(s)})$ is the CRLB when the heading angle deviation of the *s*th particle is $\eta^{t,k(s)}$ and the related position of the *s*th particle is $x^{t,k(s)}$, and Tr(·) denotes the trace operation. Then, the individual historical best position $p^{t,k+1(s)}$ and the global best position $p_{g}^{t,k+1(s)}$ can be updated as

(36) $$p^{t,k+1(s)}=\left\{\begin{array}{cc} \eta^{t,k+1(s)} & \mathrm{for}\;Fn\left(x^{t,k+1(s)}\right)<Fn\left(p^{t,k(s)}\right)\\ p^{t,k(s)} & \mathrm{else} \end{array}\right.$$

(37) $$p_{g}^{t,k+1}=\underset{1\leq s\leq S}{arg min}Fn\left(p^{t,k+1(s)}\right)$$

**Figure 1:**
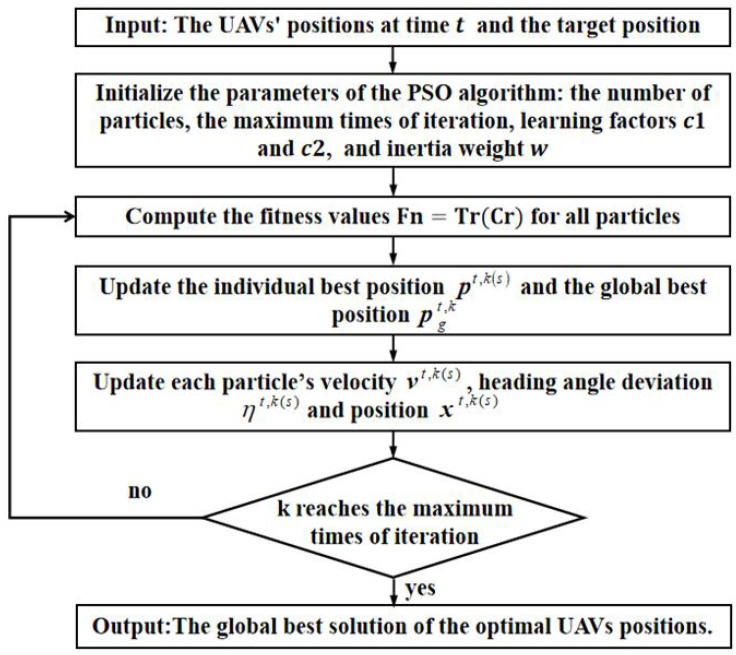
The flowchart of the PSO algorithm.

When the iteration reaches the maximum number, the iteration stops and outputs the optimal solution; thus, the optimal UAV positions for the next time step (t+1) can be obtained. The flowchart of the PSO algorithm is shown as follows, see Figure 1.

## 5. Numerical Simulations and Results

In this section, numerical simulations are performed to verify the validity and effectiveness of the proposals in this paper. Firstly, the basic parameters and the initial topology of the UAVs and the target are given. Secondly, five methods are presented and compared to verify the advantages of the proposed method in this paper, and the five methods include the following: (1) the PSO algorithm considering the UAVs to have variable speed; (2) the PSO algorithm considering the UAVs to have a fixed speed; (3) the exhaustive algorithm considering the UAVs to have variable speed; (4) the exhaustive algorithm considering the UAVs to have a fixed speed; (5) the algorithm considering the UAVs to have a fixed formation.

### 5.1. Parameters Configuration

**Figure 2:**
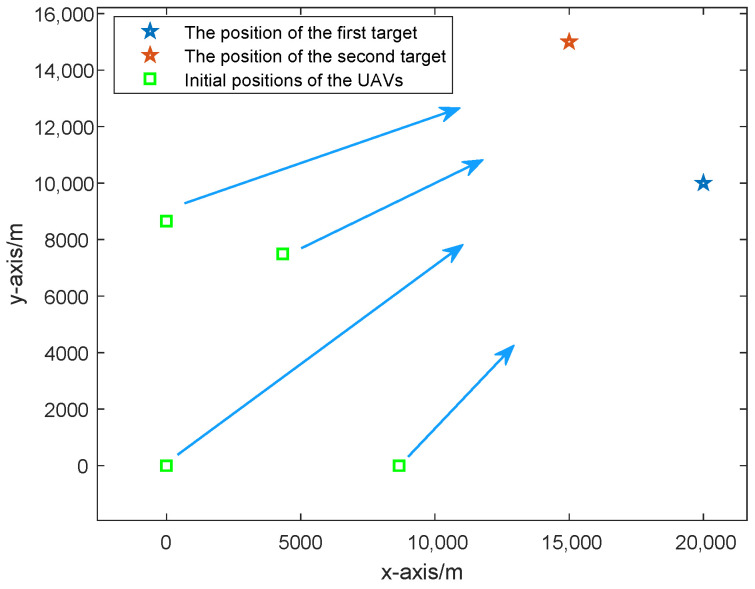
The initial topology of the UAVs and the targets.

Consider that there are four UAVs and two stationary targets in a two-dimensional plane. The initial coordinates of the UAVs are [0, 0] m, [8660, 0] m, [4330, 7500] m, and [0, 8660] m, and the targets are located at [20,000, 10,000] m and [15,000, 15,000] m. The initial topology of the UAVs and the targets is given in Figure 2, where the green squares represent the initial positions of the UAVs, the blue and red five-pointed stars indicate the two target positions, and the blue arrows indicate the moving directions of the UAVs.

Through systematic parameter sensitivity analysis, we have determined the optimal parameter configuration scheme in this paper. The speed range for the variable-speed UAVs is set to [200, 400] m/s, and the speed value for the fixed-speed UAVs is set to 300 m/s. Through systematic experiments, it is determined that the global optimal fitness value stabilizes when the number of iterations reaches 110. To ensure algorithmic robustness, the maximum number of iterations is ultimately set to 150. The cognitive component c1 and the social component c2 are both set to 2 to effectively balance the influence of individual particle experience and swarm knowledge on the search process, and the inertia weight *w* is set to 0.9 to maintain strong global search capability during the initial phase of the algorithm. Since the feasible region for the PSO algorithm considering the UAVs with variable speed is larger than that for the PSO algorithm considering the UAVs with fixed speed, the number of the particles is 1000 for the PSO algorithm considering the UAVs with variable speed and the number of the particles is 100 for the PSO algorithm considering the UAVs with fixed speed, which can balance computational efficiency with precision requirements. To mitigate collision risks, the minimum approach distance is set to 8000 m, the heading angle range is set to [−π/4, π/4], and the heading deflection angle range is set to [−π/180,π/180].

### 5.2. Simulation Results Based on the Exhaustive Algorithm

**Figure 3:**
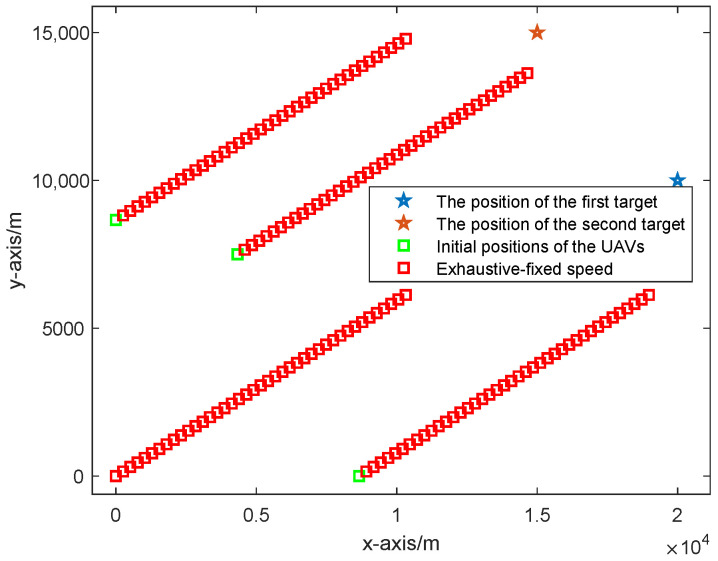
UAV path based on the fixed formation algorithm.

**Figure 4:**
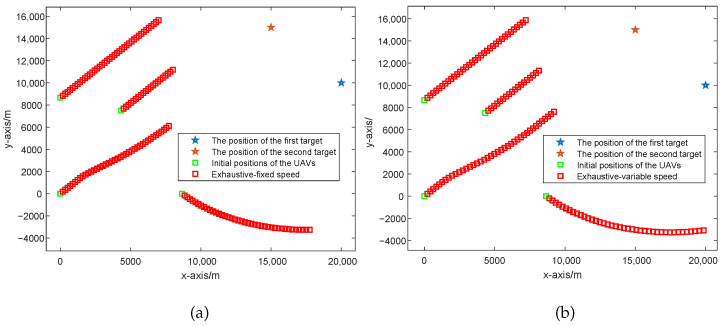
UAV paths optimized by the exhaustive algorithm. (**a**) UAV path optimized considering the UAVs to have a fixed speed. (**b**) UAV path optimized considering the UAVs to have variable speed.

In this part, we conduct the simulation experiments for different UAV path optimization algorithms, which include (1) UAVs with a fixed formation; (2) the UAV path optimization algorithm based on CRLB considering UAVs with fixed speed; and (3) the UAV path optimization algorithm based on CRLB considering UAVs with variable speed. The simulation results of the UAV path based on different UAV path optimization methods are given in Figure 3 and Figure 4, where Figure 3 shows the UAV path with a fixed formation and Figure 4 shows the UAV paths optimized by the CRLB algorithm where (a) considers UAVs with a fixed speed and (b) considers the UAVs with variable speed. Also, in Section 5.2, we consider the UAV path optimized based on the exhaustive algorithm.

To evaluate the localization accuracy of different methods, we employ the Root Mean Square Error (RMSE) as the accuracy metric and conduct a comparative analysis of the localization accuracy between different methods.

(38) $$\mathrm{RMSE}\left(k\right)=\sqrt{\frac{1}{N}\sum_{i=1}^{N}{\left(u_{k}-{\hat{u}}_{k}\right)}^{2}}$$

where *N* denotes the number of Monte Carlo experiment trials, $u_{k}$ represents the true position of the target at time step *k* and ${\hat{u}}_{k}$ represents the estimated position of the target at time step *k*. A total of 100 independent Monte Carlo trials are conducted to complete the comparison.

**Figure 5:**
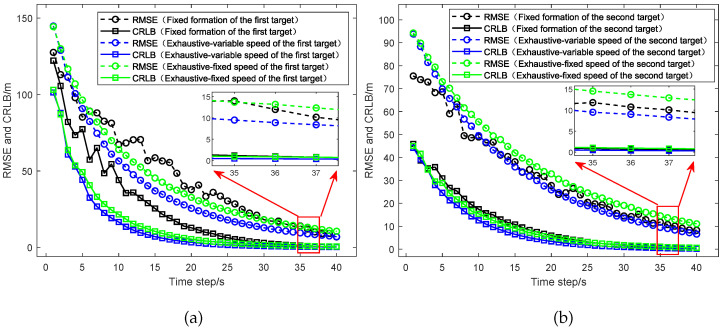
Comparison of the RMSE and CRLB between different UAV optimization algorithms which include the fixed formation algorithm, the exhaustive algorithm considering the UAVs with fixed speed, and the exhaustive algorithm considering the UAVs with variable speed. (**a**) Performance comparison for the target at [20,000, 10,000] m. (**b**) Performance comparison for the target at [15,000, 15,000] m.

From Figure 5, the simulation results of the RMSE and CRLB between different UAV path optimization algorithms in the dual-target scenario are shown and compared, including the fixed formation, the exhaustive algorithm considering UAVs with fixed speed, and the exhaustive algorithm considering UAVs with variable speed. (a) presents the comparison between the RMSE and CRLB for the target located at [20,000, 10,000] m, while (b) shows the comparison between the RMSE and CRLB for the target located at [15,000, 15,000] m. From the results, we can see that for the target located at [20,000, 10,000] m, the RMSE of the UAVs with fixed formation converges to 12.23 m and the CRLB converges to 0.90 m. The RMSE of the UAV path optimized based on the exhaustive algorithm considering the UAVs with fixed speed converges to 14.47 m and the CRLB converges to 0.89 m. The RMSE of the UAV path optimized based on the exhaustive algorithm considering the UAVs with variable speed converges to 9.85 m and the CRLB converges to 0.41 m. For the target located at [15,000, 15,000] m, the RMSE of the UAVs with fixed formation converges to 11.65 m and the CRLB converges to 0.47 m. The RMSE of the UAV path optimized based on the exhaustive algorithm considering the UAVs with fixed speed converges to 15.21 m and the CRLB converges to 0.98 m. The RMSE of the UAV path optimized based on the exhaustive algorithm considering the UAVs with variable speed converges to 9.65 m and the CRLB converges to 0.40 m. Thus, compared to the UAVs with fixed formation, the UAV path optimized based on the exhaustive algorithm considering the UAVs with variable speed reduces the average RMSE of the two targets by about 18.35% and reduces the average CRLB by about 50.16%. Then, compared to the UAV path optimized based on the exhaustive algorithm considering the UAVs with fixed speed, the UAV path optimized based on the exhaustive algorithm considering the UAVs with variable speed reduces the average RMSE by about 34.27% and reduces the average CRLB by about 56.62%. The results indicate that by considering the UAVs with variable speed, the UAV path optimization algorithm could significantly improve the target localization accuracy in the dual-target scenario.

### 5.3. Simulation Results Based on the PSO Algorithm

In Section 5.2, the simulation results for the UAV path optimization algorithms, which include the fixed formation, the UAV path optimization algorithm considering the UAVs with fixed speed, and the UAV path optimization algorithm considering the UAVs with variable speed, are all obtained based on the exhaustive algorithm. To improve the effectiveness of the path optimization algorithm, in this paper, the PSO algorithm is deployed to search for the optimal positions of the UAVs at the next time step, reducing the computation complexity and improving the real-time performance.

**Figure 6:**
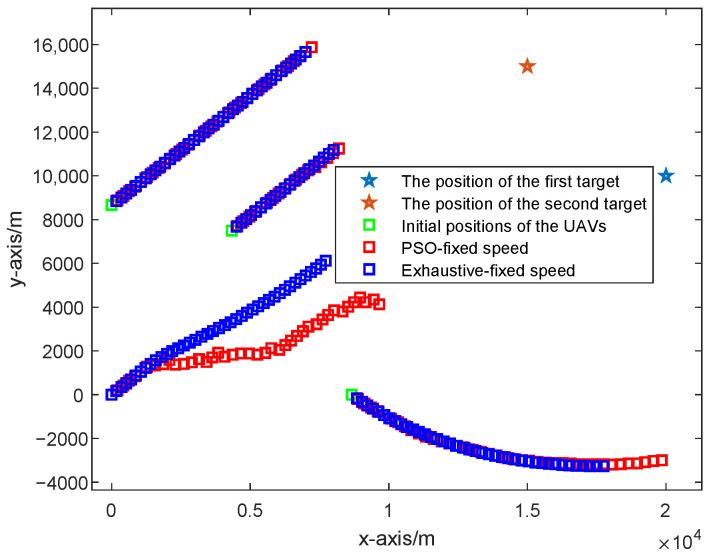
Different UAV paths optimized based on the PSO algorithm and the exhaustive algorithm considering the UAVs with fixed speed.

As can be seen from Figure 6, the UAV paths optimized based on the PSO algorithm and the exhaustive algorithm considering the UAVs with fixed speed are shown and compared, where the red squares represent the UAV path optimized based on the PSO algorithm, and the blue squares represent the UAV path optimized based on the exhaustive algorithm. Obviously, the UAV paths optimized based on the PSO algorithm and the exhaustive algorithm considering the UAVs with fixed speed are almost the same because they are both optimized based on the CRLB criterion but by different optimization algorithms.

**Figure 7:**
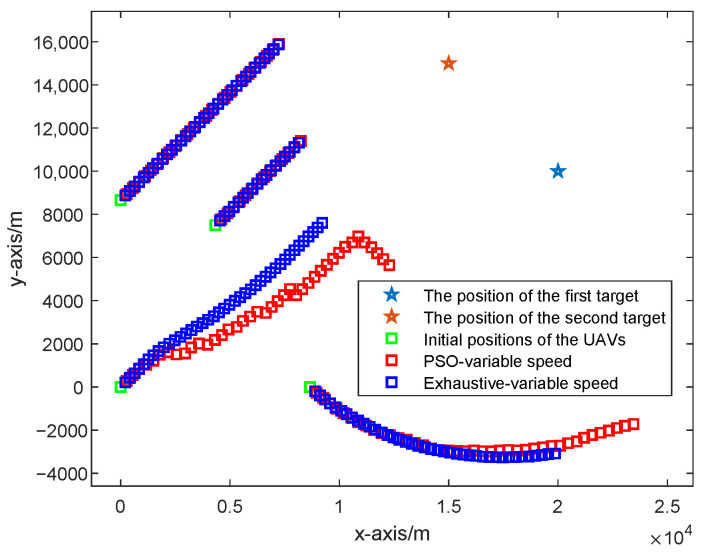
Different UAV paths optimized based on the PSO algorithm and the exhaustive algorithm considering the UAVs with variable speed.

As shown in Figure 7, the UAV paths optimized based on the PSO algorithm and the exhaustive algorithm considering the UAVs with fixed speed are shown and compared, where the red squares represent the UAV path optimized by the PSO algorithm considering the UAVs with variable speed, and the blue squares represent the UAV path optimized by the exhaustive algorithm considering the UAVs with variable speed. Obviously, the UAV paths optimized based on the PSO algorithm and the exhaustive algorithm considering the UAVs with variable speed are almost the same, because they are both optimized based on the CRLB criterion but by different optimization algorithms.

**Figure 8:**
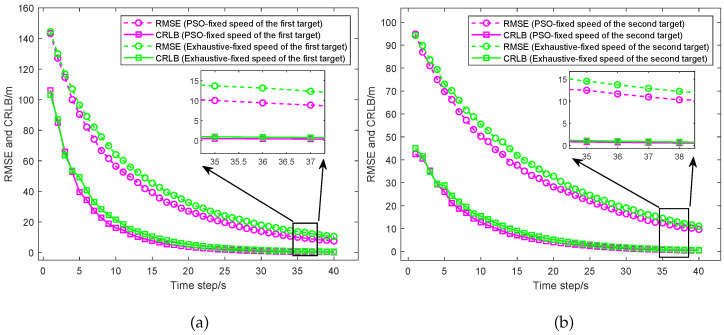
The RMSE and the CRLB based on the PSO algorithm and the exhaustive algorithm considering the UAVs with fixed speed. (**a**) Performance comparison for the target at [20,000, 10,000] m. (**b**) Performance comparison for the target at [15,000, 15,000] m.

As shown in Figure 8, the RMSE and the CRLB for different UAV paths optimized by the PSO algorithm and the exhaustive algorithm considering UAVs with fixed speed are given and compared. (a) presents the comparison between the RMSE and CRLB for the target located at [20,000, 10,000] m, while (b) shows the comparison between the RMSE and CRLB for the target located at [15,000, 15,000] m. From the simulation results, we can see that for the target located at [20,000, 10,000] m, the RMSE for the UAV path optimized by the exhaustive algorithm considering the UAVs with fixed speed converges to 14.47 m and the CRLB converges to 0.90 m. The RMSE for the UAV path optimized by the PSO algorithm considering the UAVs with fixed speed converges to 10.47 m and the CRLB converges to 0.46 m. For the target located at [15,000, 15,000] m, the RMSE for the UAV path optimized by the exhaustive algorithm considering the UAVs with fixed speed converges to 15.21 m and the CRLB converges to 0.98 m. The RMSE for the UAV path optimized by the PSO algorithm considering the UAVs with fixed speed converges to 13.05 m and the CRLB converges to 0.72 m. Thus, compared to the UAV path optimized based on the exhaustive algorithm considering the UAVs with fixed speed, the UAV path optimized based on the PSO algorithm considering the UAVs with fixed speed reduces the average RMSE of the two targets by about 20.93% and reduces the average CRLB by about 37.40%. The results indicate that according to the PSO algorithm considering the UAVs with fixed speed, the UAV path optimization algorithm could significantly improve the target localization accuracy in the dual-target scenario.

**Figure 9:**
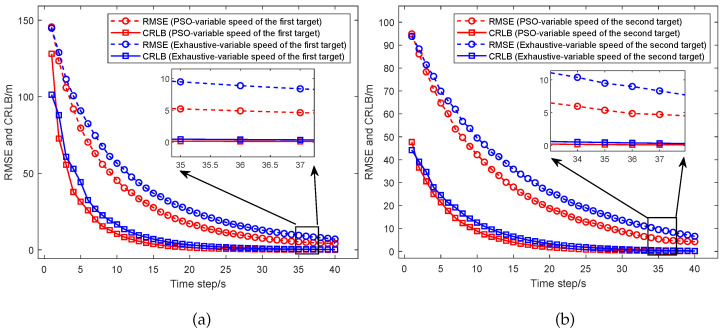
The RMSE and the CRLB based on the PSO algorithm and the exhaustive algorithm considering the UAVs with variable speed. (**a**) Performance comparison for the target at [20,000, 10,000] m. (**b**) Performance comparison for the target at [15,000, 15,000] m.

The situation where the UAVs are considered with variable speed is almost the same as the situation where the UAVs are considered with fixed speed. As shown in Figure 9a, the RMSE for the UAV path optimized by the PSO algorithm converges to 5.54 m and the CRLB converges to 0.13 m. The RMSE for the UAV path optimized by the exhaustive algorithm converges to 9.85 m and the CRLB converges to 0.41 m. As shown in Figure 9b, the RMSE for the UAV path optimized by the PSO algorithm converges to 5.62 m and the CRLB converges to 0.13 m. The RMSE for the UAV path optimized by the exhaustive algorithm converges to 9.65 m and the CRLB converges to 0.40 m. Thus, the RMSE and the CRLB obtained by the PSO algorithm are significantly lower than those obtained by the exhaustive algorithm. Compared to the UAV path optimized based on the exhaustive algorithm, the UAV path optimized based on the PSO algorithm considering the UAVs with variable speed reduces the average RMSE of the two targets by about 42.76% and reduces the average CRLB by about 67.58%. The results indicate that according to the PSO algorithm, the UAV path optimization algorithm could significantly improve the target localization accuracy in the dual-target scenario.

**Figure 10:**
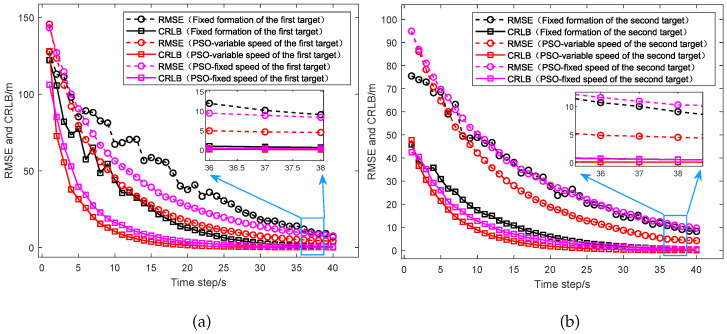
Comparison of the RMSE and CRLB between different UAV optimization algorithms which include the fixed formation, the PSO algorithm considering the UAVs with fixed speed, and the PSO algorithm considering the UAVs with variable speed. (**a**) Performance comparison for the target at [20,000, 10,000] m. (**b**) Performance comparison for the target at [15,000, 15,000] m.

As shown in Figure 10, the simulation results of the RMSE and the CRLB between different UAV path optimization algorithms are shown and compared, including the fixed formation, the PSO algorithm considering the UAVs with variable speed and the PSO algorithm considering the UAVs with fixed speed. (a) presents the comparison between the RMSE and CRLB for the target located at [20,000, 10,000] m, while (b) shows the comparison between the RMSE and CRLB for the target located at [15,000, 15,000] m. From the results, we can see that for the target located at [20,000, 10,000] m, the RMSE of the UAVs with fixed formation converges to 12.23 m and the CRLB converges to 0.90 m. The RMSE of the UAVs with fixed speed converges to 10.47 m and the CRLB converges to 0.46 m. The RMSE of the UAVs with variable speed converges to 5.54 m and the CRLB converges to 0.13 m. For the target located at [15,000, 15,000] m, the RMSE of the UAVs with fixed formation converges to 11.65 m and the CRLB converges to 0.74 m. The RMSE of the UAVs with fixed speed converges to 13.05 m and the CRLB converges to 0.72 m. The RMSE of the UAVs with variable speed converges to 5.62 m and the CRLB converges to 0.13 m. Thus, compared to the UAVs with fixed formation, the UAVs with variable speed reduce the average RMSE of the two targets by about 53.25% and the average CRLB by about 83.80%. Then, compared to the UAVs with fixed speed, the UAVs with variable speed reduce the average RMSE by about 52.03% and the average CRLB by about 77.04%. The results indicate that the PSO algorithm considering the UAVs with variable speed could significantly improve the target localization accuracy.

**Figure 11:**
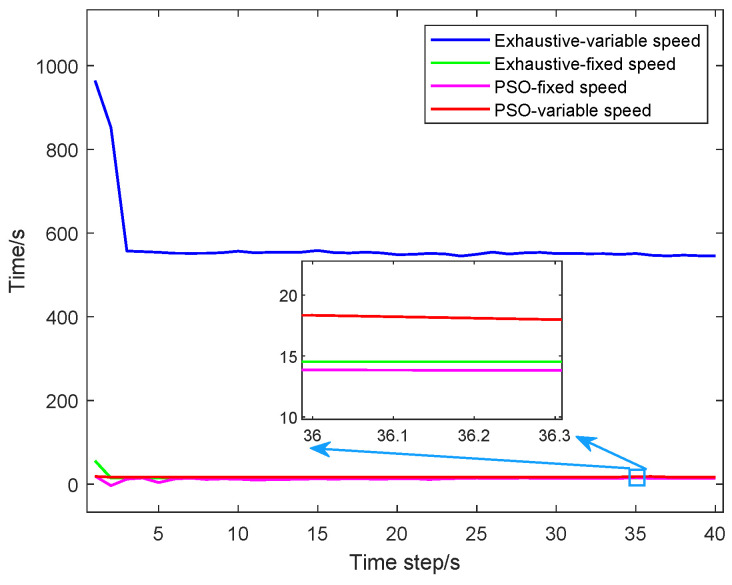
The average UAV path optimization time used for each time step for different path optimization algorithms.

To verify the effectiveness of the PSO algorithm, we compare the average computation time per time step for different UAV path optimization algorithms. From Figure 11, we can see that 17.50 s is needed for the exhaustive algorithm considering the UAVs with fixed speed, 656.44 s is needed for the exhaustive algorithm considering the UAVs with variable speed, 17.04 s is needed for the PSO algorithm considering the UAVs with fixed speed, and 20.96 s for the PSO algorithm considering the UAVs with variable speed. Thus, the computational efficiency of the PSO algorithm considering the UAVs with variable speed reduces by about 96.81% compared to the exhaustive algorithm considering the UAVs with variable speed. However, the computation time for the PSO algorithm considering the UAVs with variable speed is slightly higher than that for the PSO algorithm considering the UAVs with fixed speed and the exhaustive algorithm considering the UAVs with fixed speed. The localization accuracy of the former algorithm is better than that of the latter algorithms. Thus, from the numerical simulation results, it is illustrated that the proposed method in this paper could achieve better localization accuracy with proper algorithm efficiency.

## 6. Conclusions

In this paper, we study the problem of UAV path optimization based on the CRLB criterion. To overcome the stringent constraint for the traditional method that the UAVs have a fixed speed, in this paper, we consider that the UAVs have variable speed, which is more suitable to the real situation. However, when the UAVs have variable speed, the feasible region is extended and the computation complexity increases. Thus, in this paper, the PSO algorithm is deployed to accelerate the searching process and reduce the computation time to obtain the optimal UAV positions at the next time step. Firstly, the signal model of the target localization is established. Secondly, the CRLB for the TDOA localization algorithm is derived and the UAV path optimization problem based on the CRLB is formulated. Thirdly, the PSO algorithm is deployed to search for the optimal UAV positions for the next time step. Finally, numerical simulation results show that compared to the existing UAV path optimization algorithm, the method proposed in this paper could achieve higher localization accuracy and lower computation complexity.

## 7. Future Work

Future research will focus on a comprehensive and in-depth exploration of several key directions.

First, in terms of model extension, current studies primarily concentrate on two-dimensional space. Future work aims to extend the model to three-dimensional domains, constructing novel localization models that incorporate the altitude dimension. This will fully account for UAVs’ unique motion characteristics in 3D space to more accurately describe their positional information. Building upon this, emphasis will be placed on addressing the dynamic decision-making problem of optimal UAV flight altitude. In real-world operations, a UAV’s flight altitude is influenced by multiple factors, including obstacle distribution, signal strength, and meteorological conditions. By integrating advanced optimization algorithms with real-time environmental data, the flight altitude can be dynamically adjusted to balance localization accuracy with flight safety, energy consumption, and other multi-objective requirements. Simultaneously, a combined approach of theoretical analysis and simulation verification will be adopted to quantitatively investigate the nonlinear relationship between flight altitude and localization accuracy. An accurate mathematical model will be established, and extensive simulation experiments will be conducted to analyze the variation patterns of localization accuracy under different flight altitudes. This will provide solid theoretical guidance for 3D path optimization in complex scenarios. Furthermore, as the model incorporates more practical factors, computational complexity will increase significantly. Therefore, a quantitative analysis of computational complexity will be performed to serve as a reference for algorithm optimization and real-world applications.

In terms of algorithmic comparison and validation, we plan to establish a comprehensive multi-dimensional evaluation system. This system is designed to conduct a systematic comparison between the proposed algorithm and mainstream approaches. These mainstream approaches encompass the extended Kalman filter, genetic algorithms, and emerging deep reinforcement learning methods. The comparison will be carried out across key metrics, namely localization accuracy, computational efficiency, energy consumption characteristics, and robustness. To ensure the objectivity and accuracy of the evaluation results, scientifically justified evaluation metrics and well-designed testing scenarios will be developed. Leveraging this framework, a detailed comparative analysis will be conducted to assess algorithm performance under different conditions, thoroughly investigating the strengths, weaknesses, and applicable domains of each method. The findings will provide a theoretical basis for algorithm selection in practical applications. Additionally, targeted improvements will be proposed to address the limitations of the proposed algorithm, further enhancing its performance and competitiveness.

To better evaluate the algorithm’s practical applicability, future work also plans to conduct experiments using real UAVs. A comprehensive experimental platform will be established, integrating UAV hardware systems, sensor equipment, ground control stations, and other components to ensure smooth experiment execution. Additionally, diverse experimental scenarios will be designed, covering varying terrains, meteorological conditions, and mission requirements, to thoroughly validate the algorithm’s performance across different real-world environments. During the experiments, the focus will be on verifying the effectiveness of the proposed algorithm by comparing its performance with theoretical analyses and simulation results. Key evaluation metrics will include localization accuracy and path optimization rationality in practical applications. Furthermore, the algorithm’s computational time for determining the UAV’s next waypoint must meet real-time transmission requirements. Real-time performance serves as a critical metric for UAV applications. Only when ensuring that the algorithm can swiftly compute and transmit results can safe flight operations and mission execution be guaranteed. Experimental data analysis will further optimize the algorithm’s performance, enhancing its real-time capability and reliability.

## Data Availability

The original contributions presented in this study are included in the article. Further inquiries can be directed to the corresponding author.
